# NeoTImmuML: a machine learning-based prediction model for human tumor neoantigen immunogenicity

**DOI:** 10.3389/fimmu.2025.1681396

**Published:** 2025-10-22

**Authors:** Yan Shao, Shuguang Ge, Ruizhe Dong, Wei Ji, Chaoran Qin, Pengbo Wen

**Affiliations:** School of Medical Informatics and Engineering, Xuzhou Medical University, Xuzhou, Jiangsu, China

**Keywords:** tumor neoantigens, immunogenicity, machine learning, ensemble model, database, SHAP

## Abstract

**Introduction:**

Tumor neoantigens possess high specificity and immunogenicity, making them crucial targets for personalized cancer immunotherapies such as mRNA vaccines and T-cell therapies. However, experimental identification and evaluation of their immunogenicity are time-consuming, which limits the efficiency of vaccine development.

**Methods:**

To address these challenges, we implemented two key strategies. First, we upgraded the TumorAgDB database by integrating publicly available neoantigen data from the past two years, resulting in TumorAgDB2.0. Second, we developed NeoTImmuML, a weighted ensemble machine learning model for predicting neoantigen immunogenicity. Using data from TumorAgDB2.0, we calculated the physicochemical properties of peptides and systematically evaluated eight machine learning algorithms via five-fold cross-validation. The top-performing algorithms — LightGBM, XGBoost, and Random Forest — were integrated into a weighted ensemble model.

**Results:**

TumorAgDB2.0 (https://tumoragdb.com.cn) now contains 187,223 entries. Moreover, NeoTImmuML demonstrated strong generalization performance on both internal and external test datasets. SHAP feature importance analysis revealed that peptide hydrophilicity and length are key determinants of immunogenicity.

**Discussion:**

TumorAgDB2.0 provides a comprehensive data resource for neoantigen research, while NeoTImmuML offers an efficient and interpretable tool for predicting neoantigen immunogenicity. Together, they offer valuable support for the design of personalized neoantigen vaccines and the development of cancer immunotherapy strategies.

## 1 Introduction

In recent years, T-cell–mediated cancer immunotherapy has made remarkable progress in various solid tumors. It is now recognized as the fourth pillar of cancer treatment, following surgery, radiotherapy, and chemotherapy. At the core of this therapy is the recognition of tumor neoantigens. These are peptides generated by somatic mutations. They are tumor-specific and immunogenic. Presented by MHC molecules on tumor cells, they can trigger T-cell immune responses. Studies have shown that in patients receiving tumor-infiltrating lymphocyte (TIL) adoptive cell transfer, T cells that specifically recognize mutated neoantigens play a key role in driving effective anti-tumor responses (1). Additionally, neoantigens are closely linked to the success of immune checkpoint inhibitors (ICIs). When used as personalized vaccine targets, they have produced promising outcomes in several clinical studies (2). Despite their potential, identifying truly immunogenic neoantigens remains a major challenge. Current workflows often include high-throughput sequencing, mutation detection, and HLA-binding prediction. These methods can generate large numbers of candidate peptides (3). However, only a small portion can actually activate effective T-cell responses (4). Many studies have shown that HLA binding affinity (BA) alone is not a reliable predictor of immunogenicity. High-affinity peptides often fail to induce functional CD8^+^ T-cell responses (5). This leads to serious waste of time and resources during experimental validation. Although technologies are advancing rapidly, one major obstacle remains: it is still difficult to efficiently identify peptides with true immunogenic potential.

Current neoantigen discovery workflows still rely heavily on experimental validation. This process is time-consuming and resource-intensive (6). To overcome these challenges, machine learning (ML) has been widely applied to predict neoantigen immunogenicity. ML excels at modeling high-dimensional data and capturing nonlinear relationships (7). Recent studies have applied algorithms like k-nearest neighbors (KNN) (8), support vector machines (SVM) (9), and gradient boosting trees (XGBoost) (10) for neoantigen screening. These methods have shown promising results. They can effectively integrate the physicochemical properties, structural features, and immune-related information of peptides. This helps improve the accuracy of immunogenicity prediction (11). Moreover, several publicly available prediction tools, such as DeepImmuno (12) and DeepNeo (13), have leveraged deep learning techniques to improve neoantigen immunogenicity prediction. While these models have shown encouraging performance, their accuracy and generalizability remain constrained by the limited size and quality of available datasets. High-quality datasets have also become more available. Resources such as the TESLA (4) consortium, the National Cancer Institute (NCI) (14), and ITSNdb (15) offer reliable validation data. These datasets provide strong support for model training and independent evaluation. As a result, prediction research is moving from affinity-based approaches toward mechanism-driven modeling (16). However, the performance of ML models is still limited by data-related issues. Current public neoantigen datasets remain small in size, inconsistent in quality, and poorly integrated.

Although many cancer antigen and neoantigen peptide datasets have been published, they are scattered across different platforms and databases. Efficient integration and centralized management are lacking. Most existing databases suffer from fragmentation, narrow coverage, and limited functionality. For example, TANTIGEN2.0 focuses mainly on conventional tumor antigens (17). It lacks annotations for neoantigens and immunogenicity. dbPepNeo relies on a small number of cohort studies, which limits its usefulness for cross-cancer or multi-mutation training (18). Similarly, Neodb (19) and NEPdb (20) provide valuable resources for neoantigen collection and annotation but face challenges such as incomplete immunogenicity labeling and limited data standardization, which restrict their utility for developing robust prediction models. Additionally, the absence of standardized formats and the complexity of data cleaning further reduce model stability and generalizability. Even with rapid advances in sequencing and experimental technologies, the lack of systematic data integration remains a major bottleneck for algorithm optimization.

To fill this gap, we developed TumorAgDB2.0. This new database builds on TumorAgDB1.0. It integrates neoantigen data from the past two years and incorporates the NeoTImmuML prediction tool. TumorAgDB2.0 provides a standardized and multi-dimensional resource platform. It covers multiple cancer types, mutation categories, and immunogenicity validation results. It includes annotations for key immunogenic features, a summary of existing prediction tools, and seamless access to NeoTImmuML for fast and accurate prediction. This platform solves key issues found in earlier databases—such as scattered data, limited size, and single-function design. It offers solid data and tool support for advancing neoantigen prediction research. Using TumorAgDB2.0, we computed physicochemical features of each peptide. We then evaluated the performance of eight mainstream ML algorithms using five-fold cross-validation. Among them, LightGBM, XGBoost, and Random Forest performed the best. Based on these results, we developed a weighted ensemble learning framework called NeoTImmuML. This framework improves both prediction accuracy and stability. To enhance the model’s practical value, we applied SHAP (21). SHAP quantifies and visualizes the contribution of each feature to the model’s output. This helped identify the key determinants of immunogenicity. Finally, we tested NeoTImmuML on an external independent dataset. It outperformed all single models in both accuracy and AUROC. The model showed strong generalization and promising clinical potential. By building TumorAgDB2.0 and developing NeoTImmuML, this study provides powerful technical support for efficient neoantigen screening and personalized cancer vaccine design.

## 2 Materials and methods

### 2.1 Statistical analysis of TumorAgDB2.0 data

This study builds upon the TumorAgDB1.0 (22) database by incorporating the latest research findings to construct an updated version, TumorAgDB2.0. TumorAgDB1.0 included neoantigen immunogenicity data from several authoritative sources, such as peptide–MHC binding and T-cell epitope information from the Immune Epitope Database (IEDB) (23), experimentally validated neoantigen data from the National Cancer Institute (NCI), and 608 neoantigen sequences published by the TESLA alliance. It also integrated human cancer neoantigen data from the CADv1.0 platform, released by Yu Jijun et al. in 2022, forming a high-quality, multi-source foundational dataset (24).

TumorAgDB2.0 adds neoantigen immunogenicity data published between January 2024 and May 2025 (25, 26). All data were collected from peer-reviewed studies. We searched the PubMed database using keywords such as “immunogenicity,” “neoantigen,” “tumor,” and “neoepitope” to identify relevant studies on human cancers. Articles were first screened based on their titles, abstracts, and keywords, followed by manual review. From each eligible article, we extracted peptide data with confirmed immunogenicity and verified source reliability to ensure scientific rigor and accuracy.

**Figure 1:**
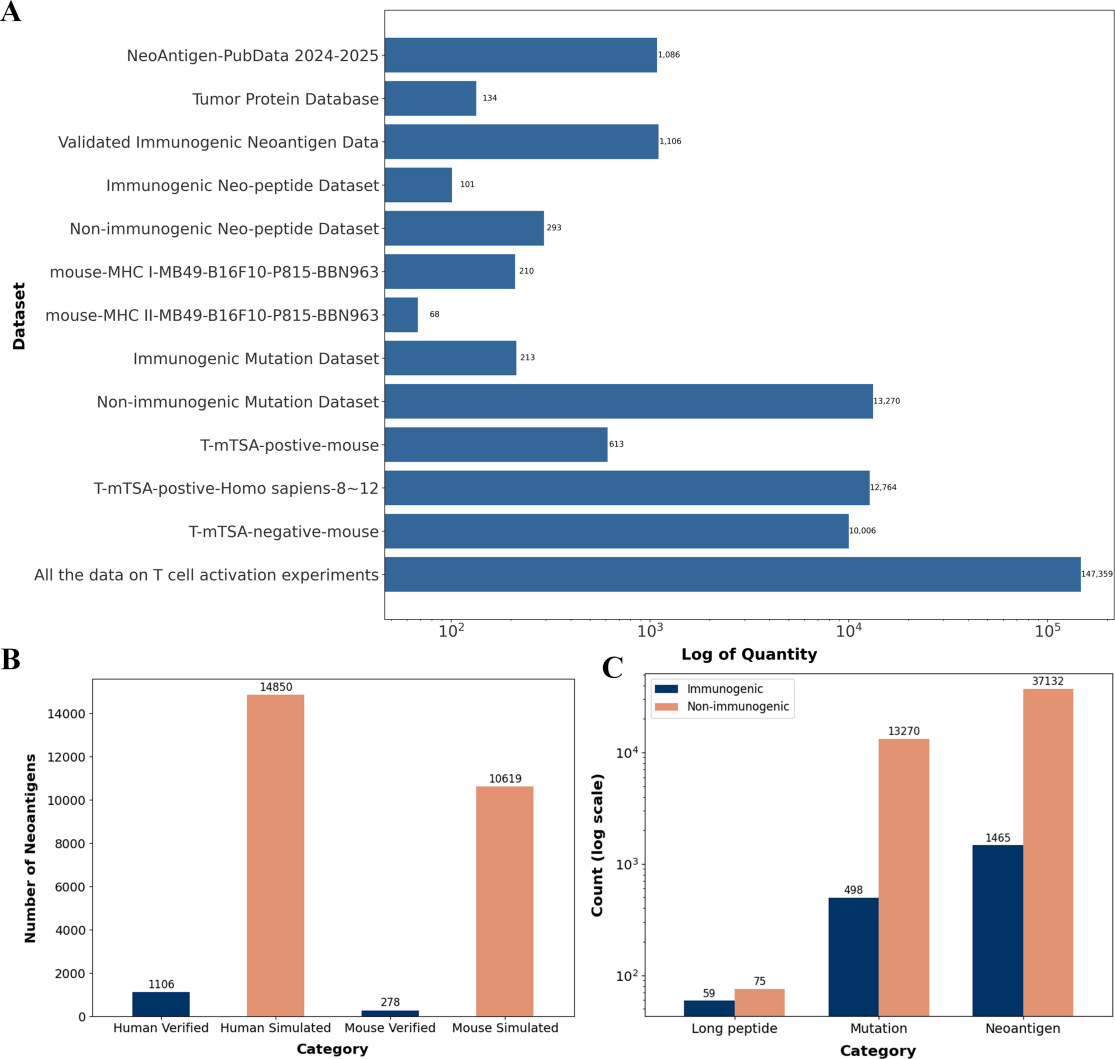
Statistical analysis of neoantigen data in TumorAgDB2.0. **(A)** Distribution of neoantigen counts across 13 categorized datasets available on the download page. **(B)** Comparison of validated and simulated neoantigens in human and mouse datasets. **(C)** Distribution of immunogenic and non-immunogenic neoantigens categorized by peptide length, including short peptides, mutant peptides, and long peptides.

The updated database now includes neoantigen data from 15 cancer types or cell lines, including colon adenocarcinoma, melanoma, invasive breast cancer, esophageal cancer, cervical cancer, cholangiocarcinoma, pancreatic adenocarcinoma, lung adenocarcinoma, renal clear cell carcinoma, gastric adenocarcinoma, sarcoma, endometrial cancer, bladder cancer, and mast cell leukemia. These data are organized into 13 functional datasets. The number of neoantigens in each dataset is shown in **Figure 1A**. All data are freely available for download.

The database contains neoantigen data derived from both human and mouse sources. Human neoantigens were validated using gold-standard immunological assays such as enzyme-linked immunospot (ELISPOT) and fluorescence-activated cell sorting (FACS). Due to the limited availability of human data, we also included mouse-validated neoantigens to expand the dataset. TumorAgDB2.0 currently contains 1,106 validated neoantigens, most of which are annotated with clear immunogenicity labels.

To improve the robustness and generalizability of machine learning models, we incorporated a large number of simulated datasets into the database (**Figure 1B**). These simulated data help address the scarcity of experimental samples and provide additional resources for training and evaluating immunogenicity prediction models. We also conducted a statistical analysis of peptide length distributions among human neoantigens (**Figure 1C**). Peptides were categorized into three groups: long peptides (>25 amino acids), mutant peptides (13–25 amino acids), and short peptides (8–12 amino acids). The results show that short peptides (8–12 amino acids) are the most prevalent. These peptides fall within the typical length range presented by MHC class I molecules and represent the primary targets in current immunogenicity prediction research.

### 2.2 Data sources used for NeoTImmuML development

In this study, we built a standardized dataset from the TumorAgDB2.0 database to develop the NeoTImmuML model. Because experimentally validated tumor neoantigen data remain limited, we also introduced simulated data. This increased the diversity and robustness of model training.

For the positive dataset, we first included peptides confirmed as immunogenic by functional assays, such as ELISPOT or flow cytometry (FACS). We then generated simulated positive peptides from human T-cell epitopes in IEDB. Mutant peptides that could trigger T-cell immune responses were selected. Only peptides with predicted binding affinity IC50 ≤ 500 nM were retained to ensure immunological relevance.For the negative dataset, we prioritized peptides verified as non-immunogenic by *in vitro* functional assays. If a peptide failed to induce CD8^+^ T cells to produce cytokines (e.g., IFN-γ, TNF-α) and no proliferation was observed, it was considered non-immunogenic (27). These data came mainly from published studies and databases such as TESLA and IEDB, where entries were explicitly labeled “non-immunogenic” with original experimental records. This helped us avoid indirect assumptions. At the same time, we generated simulated negative peptides from the NCBI dbSNP database. Nonsynonymous SNVs with high frequency (MAF > 0.05) were used to generate mutant peptides, and their wild-type counterparts were also included. To further reduce false positives, peptides with predicted IC50 ≤ 500 nM were removed. Only peptides with weaker binding were kept as negative samples.

All datasets were then standardized by deduplicated across databases, and restricted to peptide lengths of 8–13 amino acids. After strict filtering and preprocessing, we built a balanced dataset containing 5,156 positive samples (immunogenic) and 5,156 negative samples (non-immunogenic). The dataset was randomly split at an 8:2 ratio into a training set and an independent test set for model development and evaluation.

### 2.3 Feature calculation for neoantigens

To extract physicochemical property features of peptides, we used the “Peptides” package in R (version 2.4.6) (28). This tool integrates a variety of amino acid physicochemical property indices and includes 20 types of feature parameters. Each parameter can generate multiple feature values. As a result, each peptide can have up to 78 numerical features within a single feature dimension. A complete list of feature names and calculation details is provided in **Supplementary Table 1**.

### 2.4 Construction of a weighted ensemble model to enhance predictive performance

In this study, we selected eight commonly used classification models: LightGBM, XGBoost, Random Forest, Naive Bayes, Logistic Regression CV, Support Vector Machine (SVM), K-Nearest Neighbors (KNN), and Multi-Layer Perceptron (MLP). We evaluated the performance of these models using five-fold cross-validation. Preliminary results showed that LightGBM (n_estimators = 300, learning_rate = 0.05, max_depth = 7, num_leaves = 31, min_child_samples = 50, subsample = 0.6, colsample_bytree = 0.8, reg_lambda = 0.01), XGBoost (n_estimators = 200, learning_rate = 0.05, max_depth = 5, min_child_weight = 3, subsample = 0.6, colsample_bytree = 1.0, gamma = 0.1, reg_alpha = 0.01), and Random Forest (n_estimators = 300, max_depth = 7, max_features = None, min_samples_split = 2, min_samples_leaf = 4) performed well across multiple key metrics. These models demonstrated strong generalization ability. Specifically, all three achieved an AUC greater than 0.80, an accuracy above 0.70, and a precision exceeding 0.80. Their performance significantly surpassed that of the other models.

To further improve generalization and robustness, we constructed an ensemble model. The predicted probabilities from the three best-performing base classifiers were used as inputs and combined using a weighted voting strategy. To determine the optimal weight combination, we set the weight range for each model from 0 to 10 and conducted an exhaustive grid search over all possible combinations (w_1_, w_2_, w_3_ ∈ (0, 10)). For each weight combination (a, b, c), we calculated the weighted average of the predicted probabilities for the positive class (probability of class 1) from the three models, as shown in Equation 1:


(1) $$\begin{array}{c} y_{score}=\frac{\text{a}\times {\text{y}}_{1}\;+\text{b}\times {\text{y}}_{2}\;+\text{c}\times {\text{y}}_{3}\;}{\text{a}+\text{b}+\text{c}} \end{array}$$

Here, y_1_, y_2_, and y_3_ represent the predicted probabilities of the positive class from the three models. The weighted average probability was thresholded at 0.5 to perform binary classification, producing the final label. Then, we ranked all weight combinations by their AUC values. The combination with the highest AUC was selected as the final weight configuration.The best weight group was a = 4, b = 8, c = 9, which was used to build the optimal weighted ensemble classifier.

### 2.5 Evaluation of machine learning models

In this study, we employed multiple evaluation metrics to comprehensively assess the performance of various machine learning algorithms in predicting the immunogenicity of human tumor neoantigens. These metrics included accuracy (Acc), precision (Pre), recall (Recall), F1-score, specificity (Spe), and the area under the ROC curve (AUROC).

The formulas for these metrics are provided in Equations 1–6.

True Positive (TP) refers to the number of peptides correctly predicted as immunogenic.

True Negative (TN) refers to the number of peptides correctly predicted as non-immunogenic.

False Positive (FP) is the number of non-immunogenic peptides incorrectly predicted as immunogenic. False Negative (FN) is the number of immunogenic peptides incorrectly predicted as non-immunogenic.

AUROC represents the area under the ROC curve and evaluates the model’s ability to distinguish between classes across all classification thresholds. The closer the AUROC value is to 1, the better the model’s performance.

Accuracy (Acc) is the ratio of correctly classified samples to the total number of samples. It reflects the overall classification performance of the model, as shown in Equation 2.


(2) $$\begin{array}{c} \text{Acc}=\frac{\text{TP}+\text{TN}}{\text{TP}+\text{TN}+\text{FP}+\text{FN}} \end{array}$$

Precision (Pre) is the proportion of correctly predicted positive samples among all samples predicted as positive. It measures the accuracy of the model’s positive predictions, as shown in Equation 3.


(3) $$\begin{array}{c} \text{Pre}=\frac{\text{TP}}{\text{TP}+\text{FP}} \end{array}$$

Recall is the proportion of actual positive samples that are correctly identified by the model. It reflects the model’s sensitivity in detecting positive samples, as shown in Equation 4.


(4) $$\begin{array}{c} \text{Recall}=\frac{\text{TP}}{\text{TP}+\text{FN}} \end{array}$$

F1-score is the harmonic mean of precision and recall. It provides a balanced measure of both metrics, as shown in Equation 5.


(5) $$\begin{array}{c} \text{F}1-\text{score}=2\times \frac{\text{Pre}\times \text{Recall}}{\text{Pre}+\text{Recall}} \end{array}$$

Specificity (Spe) is the proportion of actual negative samples that are correctly identified as negative by the model, as shown in Equation 6.


(6) $$\begin{array}{c} \text{Spe}=\frac{\text{TN}}{\text{TN}+\text{FP}} \end{array}$$

## 3 Results

### 3.1 Overview of TumorAgDB2.0 database

**Figure 2:**
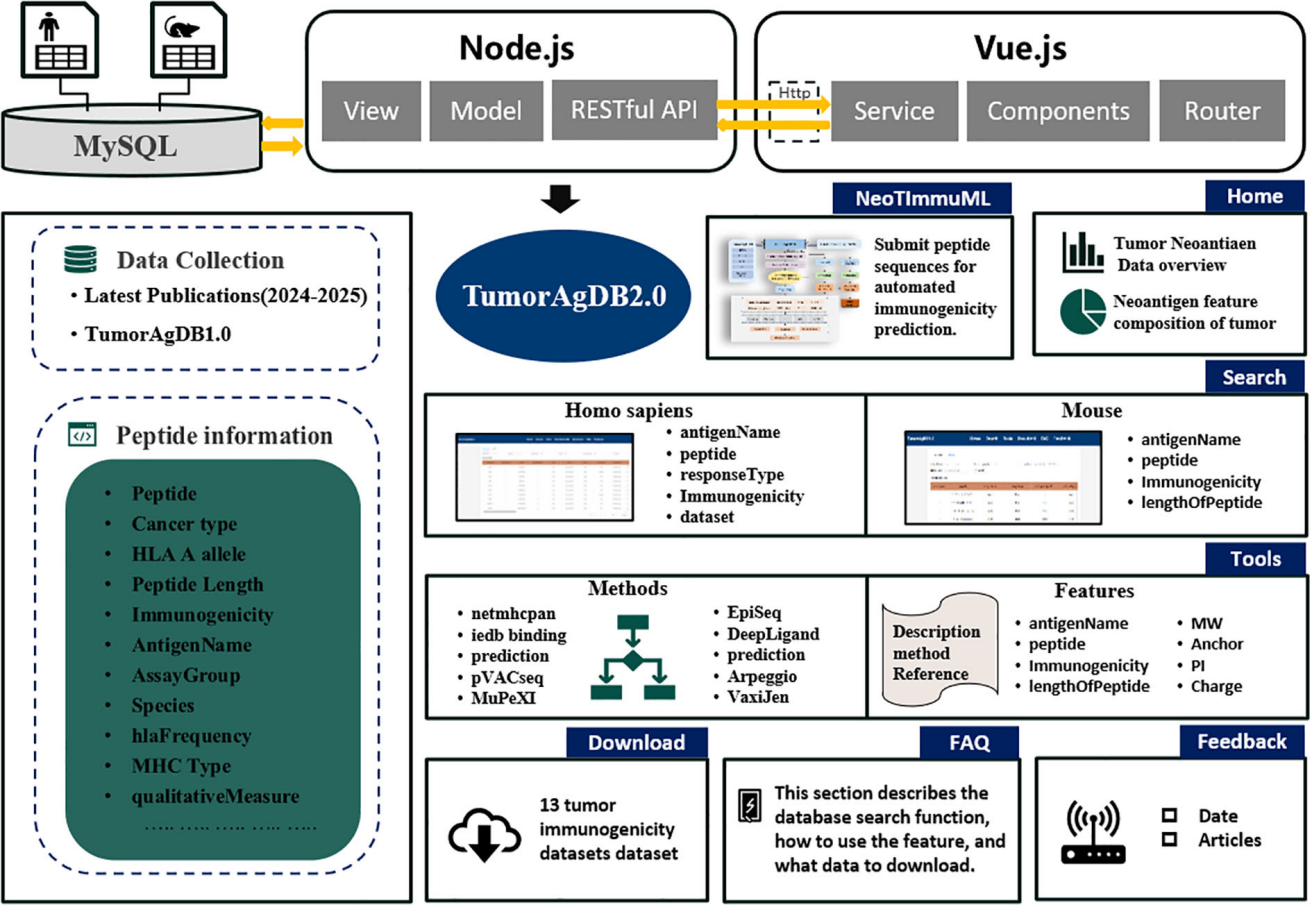
Overview of the content and construction process of the TumorAgDB2.0 database.

TumorAgDB 2.0 delivers a revamped and expanded user interface built on a modern, modular architecture. As shown in **Figure 2**, the platform now integrates seven seamless modules: (I) Home—the central entry point with an intuitive visual overview; (II) Search—for structured data retrieval; (III) Tools—hosting analytical utilities and peptide-feature calculators; (IV) NeoTImmuML—an immunogenicity-prediction interface; (V) Download—a comprehensive data repository; (VI) FAQ—step-by-step operational guidance; and (VII) Feedback—a direct user-engagement channel.

TumorAgDB2.0 provides a direct link to the NeoTImmuML GitHub repository, where users can click the corresponding card on the NeoTImmuML page to access the model code and related files. This machine-learning model predicts human tumor-neoantigen immunogenicity and achieved an average AUC of 0.8707 under five-fold cross-validation (**Supplementary Table 2**). A one-click download option provides the full source code and pretrained weights, enabling users to deploy the model locally for personalized predictions. NeoTImmuML can be accessed through its dedicated module or downloaded for offline use. The FAQ page accelerates onboarding, offering clear instructions for navigating and leveraging the platform. To maintain currency, we continuously curate peer-reviewed neoantigen-immunogenicity data published from January 2024 through May 2025. These latest datasets are available for immediate download in the Download module.

To better illustrate the practical value of TumorAgDB2.0 and NeoTImmuML, we designed a simplified workflow (**Supplementary Figure 1**). Researchers can select and download datasets from the download interface. Each dataset is accompanied by detailed descriptions to guide appropriate use. After obtaining the data, users can perform feature calculation and download our tool NeoTImmuML for model training. NeoTImmuML classifies peptides as immunogenic or non-immunogenic. Based on these predictions, researchers can prioritize peptides predicted as immunogenic for experimental validation. This helps narrow the scope of experiments and reduces unnecessary testing. The workflow demonstrates how NeoTImmuML can support experimental design, lower costs, and shorten the validation cycle.

To position TumorAgDB2.0 within the current resource landscape, we conducted a structured comparison with IEDB, NeoDB, NEPdb, dbPepNeo, and TANTIGEN2.0 (**Supplementary Table 3**). TumorAgDB2.0 is curated through May 2025 and supports open, bulk downloads. It provides feature-level annotations directly relevant to immunogenicity together with explicit computation methods, enabling reproducibility and methodological extension. The platform is stably accessible, actively maintained.

### 3.2 Performance evaluation of NeoTImmuML

**Figure 3:**
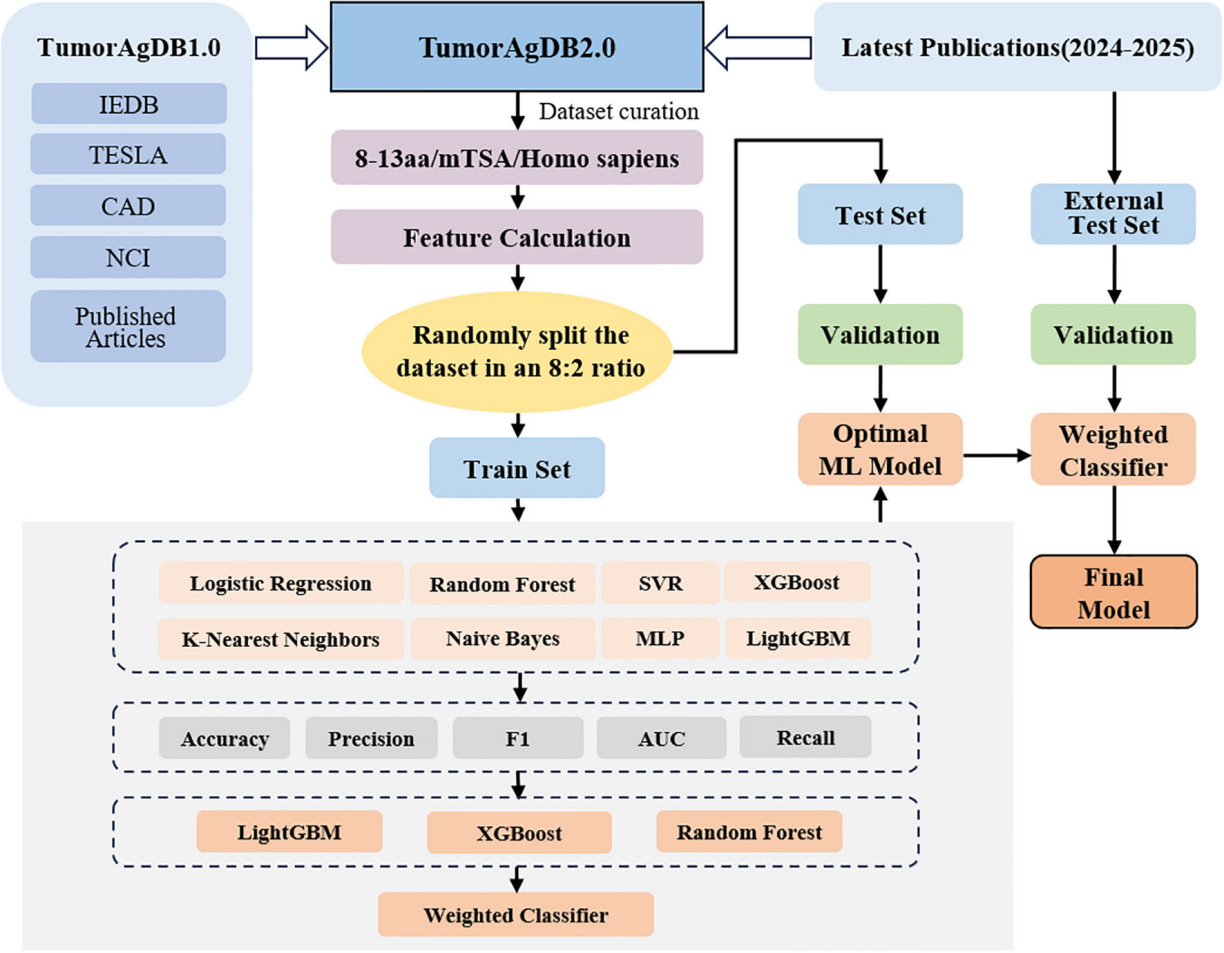
Flow chart illustrating the architecture and processing pipeline of the NeoTImmuML model.

The overall workflow for data collection, feature extraction, model evaluation, and model development is illustrated in **Figure 3**. We systematically constructed a neoantigen immunogenicity dataset by screening public databases and published literature. The final dataset contains 10,312 samples, consisting of an equal number of positive (immunogenic) and negative (non-immunogenic) examples (n = 5,156 each). We used the “Peptides” package in R to calculate physicochemical property features for each peptide sequence. These features were used as input for subsequent modeling.

During the data-splitting phase, we used the train_test_split method to randomly divide the dataset into a training set (80%) and a test set (20%), ensuring sufficient generalization capability. One-hot encoding was applied to both subsets to maintain consistent feature dimensions. We also aligned the feature structures to prevent mismatches caused by inconsistent columns. For model evaluation, we applied five-fold cross-validation on the training set and assessed the performance of eight common machine learning algorithms: LightGBM, XGBoost, Random Forest, Support Vector Classifier (SVC), Logistic Regression CV, Naive Bayes, K-Nearest Neighbors (KNN), and Multi-Layer Perceptron (MLP). In each iteration, the training data were split into five subsets. Four subsets were used for training and one for validation. This process was repeated five times so that each subset served as the validation set once. The results from all five rounds were aggregated to evaluate overall model performance. The summary of the evaluation results of each model in the cross-validation is presented in **Table 1**. LightGBM led the pack with an AUC of 0.8666, accuracy of 0.7785, precision of 0.8294, recall of 0.7034, F1 score of 0.7612, and specificity of 0.8541, while XGBoost (AUC = 0.8568) and Random Forest (AUC = 0.8522) came in close behind; every other model posted an AUC below 0.85.

**Table 1:** Performance of eight machine learning models on tumor neoantigen data.

Model	Accuracy (%)	Precision (%)	Recall (%)	F1-score (%)	AUROC (%)	Specificity (%)
LightGBM	77.85	82.94	70.34	76.12	86.66	85.41
XGBoost	77.19	80.34	72.25	76.08	85.68	82.18
Random Forest	76.20	80.09	69.98	74.70	85.22	82.47
Logistic Regression CV	75.76	79.15	70.22	74.42	83.66	81.35
Naive Bayes	76.13	81.81	67.44	73.93	84.96	84.88
MLP	70.68	72.40	67.23	69.72	78.07	74.17
SVC	68.65	71.16	63.12	66.90	76.31	74.22
K-Nearest Neighbors	66.68	68.15	63.14	65.55	72.21	70.25

**Figure 4:**
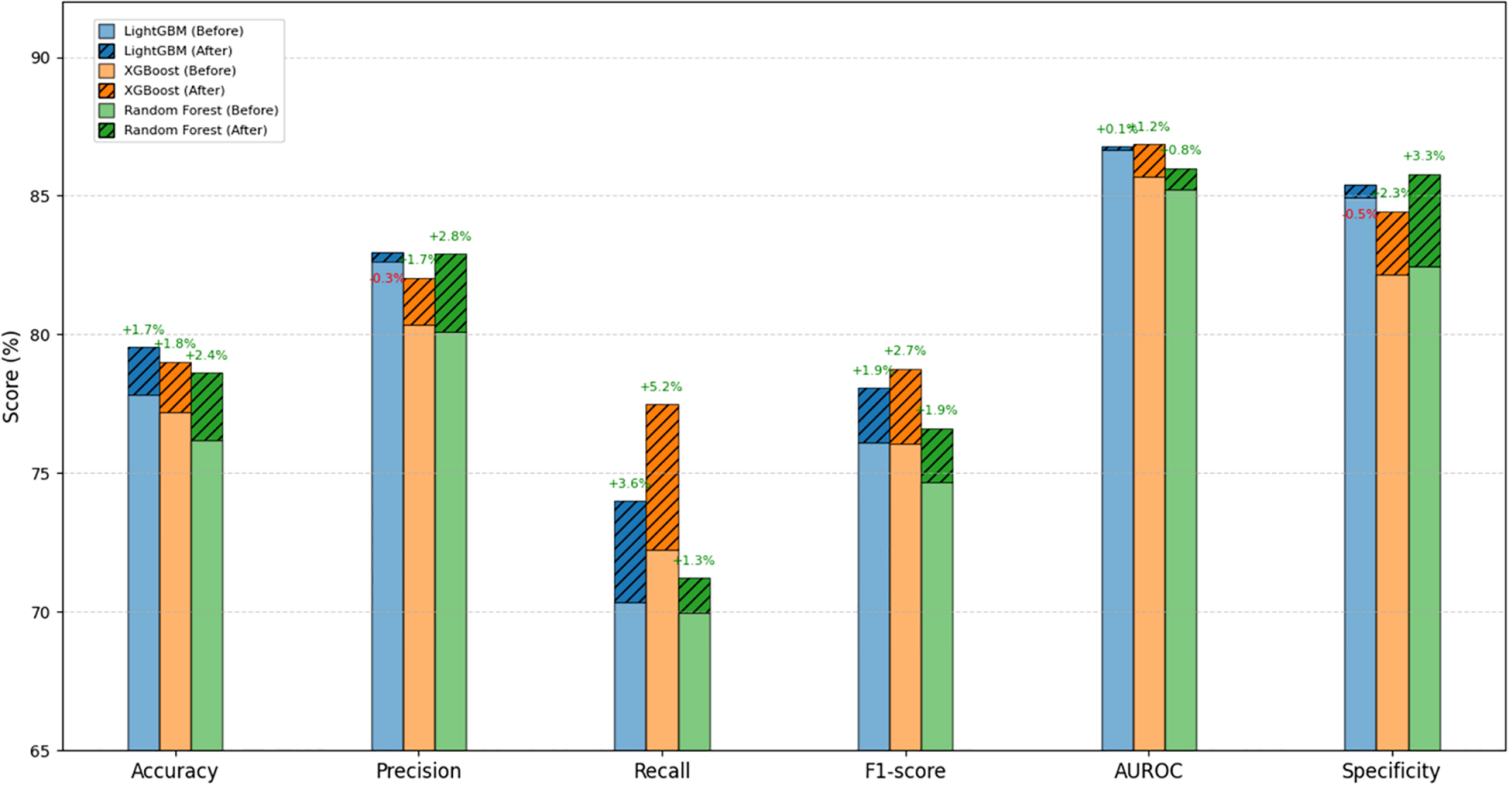
Comparison of evaluation metrics for the three machine learning models (LightGBM, XGBoost, and Random Forest) before and after parameter tuning.

**Figure 5:**
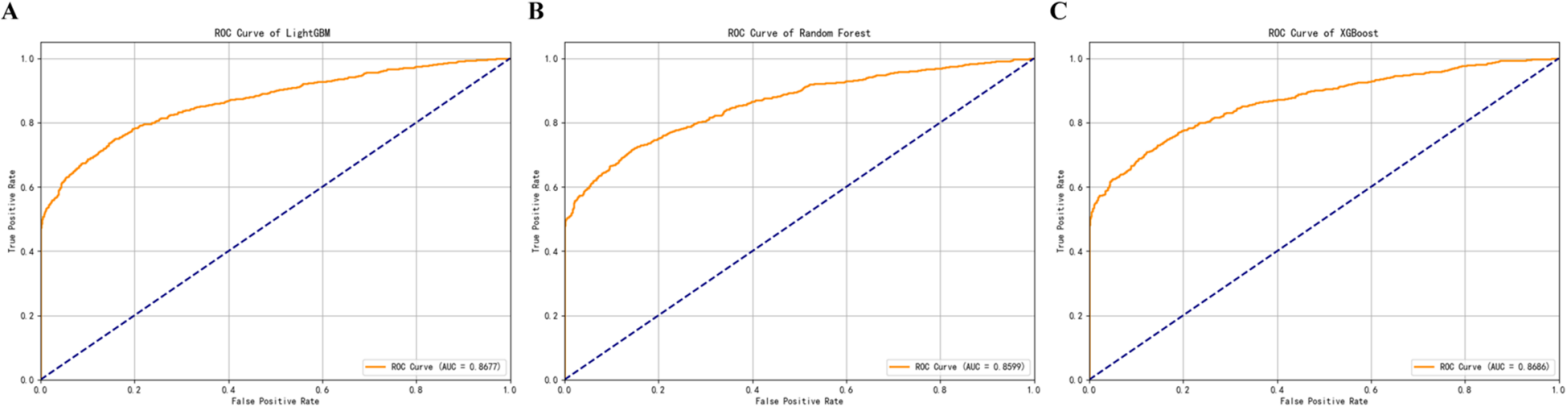
Area under the curve (AUC) values of LightGBM, XGBoost, and Random Forest models used for neoantigen immunogenicity prediction.

We then applied grid search to optimize hyperparameters for the top three models—LightGBM, XGBoost, and Random Forest. The optimized models showed improved performance across several metrics. The changes are shown in **Figure 4**, with detailed results provided in **Supplementary Table 2**. Next, we built both voting and weighted ensemble classifiers and compared their performance. The weighted ensemble model achieved an AUC of 0.8707 on the test set, slightly outperforming the voting ensemble (AUC = 0.8704) and all individual base models. This strategy improved overall performance while preserving the strengths of each base learner. Detailed performance metrics are listed in **Supplementary Table 2**, and the average ROC curves are presented in **Figures 5A–C**.

### 3.3 Independent test set validation of the model

To assess the generalization of NeoTImmuML, we built a new independent test set (n = 1,086). It was constructed by a systematic search of studies published in 2024–2025 in PubMed and CNKI. To ensure independence, we performed strict sequence-level de-duplication. We removed all peptides that overlapped with the training data. The test set is therefore completely independent of the training set.

We then conducted a head-to-head comparison on this test set. NeoTImmuML was evaluated against VaxiJen, the IEDB Class I immunogenicity predictor, and DeepImmuno. We reported AUC (threshold-independent discrimination) and F1 score (precision–recall trade-off; decision performance). NeoTImmuML achieved the best AUC (0.8865) and also showed a competitive F1 score (see **Supplementary Figure 1**). These results demonstrate robust predictive performance on unseen data.

### 3.4 SHAP-based feature importance analysis of random forest, LightGBM, and XGBoost models

To systematically analyze the contribution of physicochemical properties to neoantigen immunogenicity prediction, we applied SHAP for model interpretability (29). We performed SHAP-based analysis on the three base models—Random Forest, LightGBM, and XGBoost—within the ensemble learning framework. SHAP values were computed for each feature, and their distributions were analyzed to identify key contributors and understand how they influenced the model’s decision-making process.

**Figure 6:**
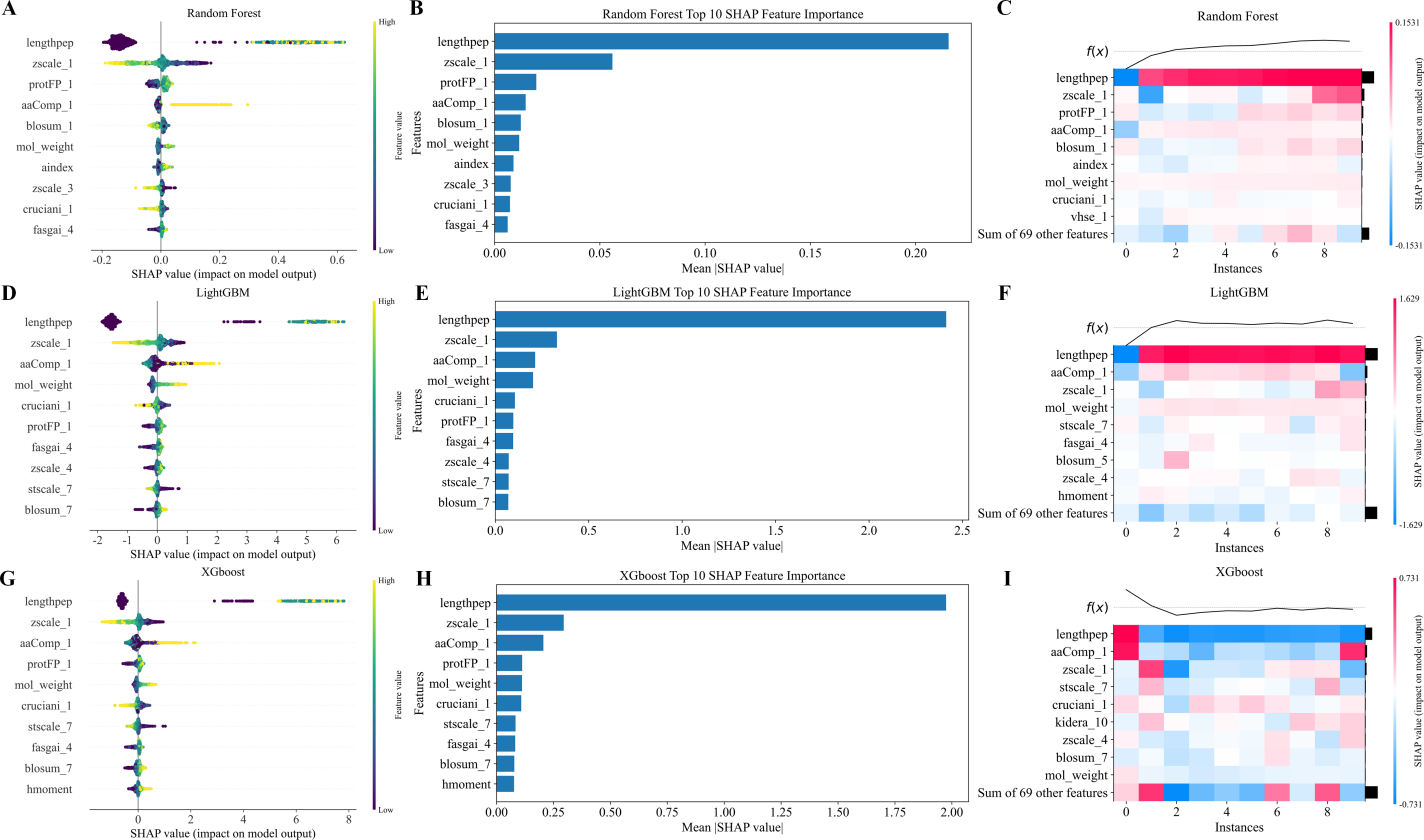
SHAP feature contribution visualization of Random Forest, LightGBM, and XGBoost models. Panels **(A–I)** show the SHAP analysis results for Random Forest, LightGBM, and XGBoost models, respectively. **(A, D, G)** display SHAP value distributions of features, illustrating their impact on model output. **(B, E, H)** present the top 10 SHAP feature importances with average contributions. **(C, F, I)** show heatmaps depicting feature influence patterns across samples.

The results showed that lengthpep (peptide length) was the most predictive feature across all three models. Its average SHAP value far exceeded those of other features (**Figures 6B, E, H**). SHAP summary plots (**Figures 6A, D, G**) indicated that longer peptides were associated with higher SHAP values, increasing the likelihood of being predicted as immunogenic. This finding aligns with known biological mechanisms, where peptides of appropriate length are more likely to form stable MHC–peptide complexes and elicit T-cell-mediated immune responses.

Following lengthpep, zscale_1 (lipophilicity) and aaComp_1 (non-polar amino acid ratio) consistently ranked among the top five features across all models. These features exhibited positively skewed SHAP distributions, suggesting that peptides with higher hydrophobicity and a greater proportion of non-polar residues are more likely to be immunogenic. Hydrophobic residues enhance peptide–MHC binding affinity, improving antigen presentation. Heatmaps (**Figures 6C, F, I**) showed consistent positive contributions of these features across samples, confirming their generalizability and biological relevance. Enhancing hydrophobicity and increasing non-polar content may improve neoantigen immunogenicity by optimizing MHC binding.

Although the three models showed strong agreement on core features, they differed in their sensitivity to secondary features. In the Random Forest model (**Figures 6A–C**), blosum_1 (sequence conservation) ranked third, suggesting that conserved amino acid sequences may promote immune recognition by maintaining structural stability. Features like aindex (amino acid index), vhse_1 (electronic properties), and mol_weight (molecular weight) were ranked lower but still contributed in certain samples.

In the LightGBM model (**Figures 6D–F**), the model showed greater dependence on mol_weight and cruciani_1 (polarity). SHAP dependency plots revealed that the contribution of these features increased notably when peptide size or polarity exceeded specific thresholds. In the XGBoost model (**Figures 6G–I**), lengthpep, zscale_1, and aaComp_1 remained dominant. XGBoost also showed higher sensitivity to features like protFP_1 (protein fingerprint), mol_weight, and hMoment (dipole moment), reflecting its strength in capturing complex, multi-dimensional physicochemical patterns.

A comparative analysis across the three models confirmed that lengthpep, zscale_1, and aaComp_1 consistently ranked among the top features, with stable contribution directions across all models. This highlights their role as core drivers of neoantigen immunogenicity. Model-specific differences revealed that Random Forest emphasized sequence conservation, LightGBM captured physicochemical thresholds, and XGBoost was more sensitive to electronic properties. These complementary perspectives underscore the strength of ensemble learning in modeling complex feature interactions.

Overall, SHAP-based interpretability analysis identified peptide length, hydrophobicity, and non-polar amino acid composition as key determinants of immunogenicity. It also enhanced the model’s transparency and interpretability, providing useful insights for experimental validation—especially in the selection and design of neoantigen peptides with optimal length and hydrophobic profiles.

## 4 Discussion

In personalized cancer immunotherapy, accurately identifying immunogenic neoantigens is essential. Traditional methods primarily rely on Major Histocompatibility Complex (MHC) binding affinity to predict immunogenicity (30). However, MHC presentation is a necessary but not sufficient condition for triggering effective immune responses. Notably, wild-type peptides can also be presented by MHC molecules. In addition, overly strong or prolonged MHC–peptide binding may lead to T-cell exhaustion. Further complicating this issue, thymic negative selection eliminates T-cell receptors that recognize both neoantigens and structurally similar wild-type peptides via central tolerance, thereby reducing the immune system’s ability to detect tumor antigens (31, 32). Therefore, relying solely on MHC binding affinity to assess immunogenicity is insufficient. An integrated approach incorporating additional biological characteristics is essential.

Machine learning offers a powerful solution by integrating multidimensional data such as amino acid physicochemical properties, MHC binding affinity, and immune-relevant features (33). This enables the modeling of complex, nonlinear relationships with immunogenicity and supports automated prediction of intricate biological processes. Motivated by this potential, we developed NeoTImmuML, a machine learning framework designed to predict neoantigen immunogenicity. Built upon the upgraded TumorAgDB2.0 database, NeoTImmuML calculates 78 features capturing physicochemical, structural, and biochemical properties of each peptide. These features represent both intrinsic sequence characteristics and biologically relevant factors linked to immunogenicity.

Recognizing the limitations of single machine learning algorithms, we evaluated eight widely used models during training using five-fold cross-validation. LightGBM, XGBoost, and Random Forest consistently outperformed the others based on accuracy and AUC. We then constructed two ensemble models—voting and weighted integration—and found that the weighted ensemble significantly outperformed both individual base learners and the voting model. Validation on an independent test set confirmed NeoTImmuML’s strong generalization ability and high predictive performance.

To improve interpretability, we used SHAP to analyze feature importance (34). Results revealed that peptide hydrophobicity and length were critical factors for immunogenicity prediction. Each algorithm emphasized different aspects: Random Forest highlighted sequence conservation features (35), LightGBM captured threshold effects of physicochemical properties (36), and XGBoost placed greater weight on electronic properties (29). This diversity illustrates the complementarity of the base models in capturing complex feature interactions and highlights NeoTImmuML’s strength in identifying biologically meaningful predictors from multiple perspectives.

NeoTImmuML shows strong performance in identifying neoantigens related to tumor-specific immunity. It provides an important theoretical basis for designing personalized immunotherapy. However, some limitations remain. The current model mainly integrates peptide-level and publicly available biological information. It does not yet include the complex immune regulatory dynamics of the tumor microenvironment (37). In addition, the dataset splitting strategy is based on sequence uniqueness rather than sequence similarity. This may introduce a risk of sequence-related bias. Future work will consider similarity-based splitting methods to improve robustness and generalization. Although TumorAgDB2.0 has expanded significantly in both data scale and dimensions compared with the previous version, the diversity and size of the training data are still limited. This may affect the model’s generalization to novel types of neoantigens (38).

It is worth emphasizing that TumorAgDB2.0 is designed as a comprehensive and continuously updated resource platform. In addition to searchable peptide information, the database integrates physicochemical features related to immunogenicity and their computational methods, an overview of current prediction tools, downloadable datasets, and literature highly relevant to immunogenicity prediction. In the future, we plan to introduce interactive visualization modules, real-time statistical analysis of search results, and direct user prediction functions. These updates will transform TumorAgDB2.0 from a static database into an interactive, user-friendly, and feature-rich platform for neoantigen research. We believe this stepwise development strategy will ensure scientific rigor while continuously enhancing the platform’s practical value and user experience.

## Data Availability

The datasets presented in this study can be found in online repositories. The names of the repository/repositories and accession number(s) can be found below: TumorAgDB2.0 (https://tumoragdb.com.cn).

## Supplementary material

Supplementary Figure 1 Schematic workflow of immunogenicity prediction using TumorAgDB2.0 and NeoTImmuML. Users can utilize their own data or data from the TumorAgDB2.0 database, and select the desired peptide features on the “Features” page to calculate physicochemical properties. Subsequently, our NeoTImmuML tool can be used to predict immunogenicity, and peptides predicted as “Immunogenic” can be considered as candidate peptides for further experimental validation.

Supplementary Figure 2 Comparison of predictive performance among NeoTImmuML, Deeplmmuno, IEDB, and Vaxijen. **(A)** Comparison of AUC values across the four prediction tools; **(B)** Comparison of F1 scores across the four prediction tools.
