# Supplementary figures and images for: NeoTImmuML: a machine learning-based prediction model for human tumor neoantigen immunogenicity
Source: Front Immunol. 2025 Oct 22;16:1681396. doi: 10.3389/fimmu.2025.1681396 (PMC12585993; doi:10.3389/fimmu.2025.1681396)

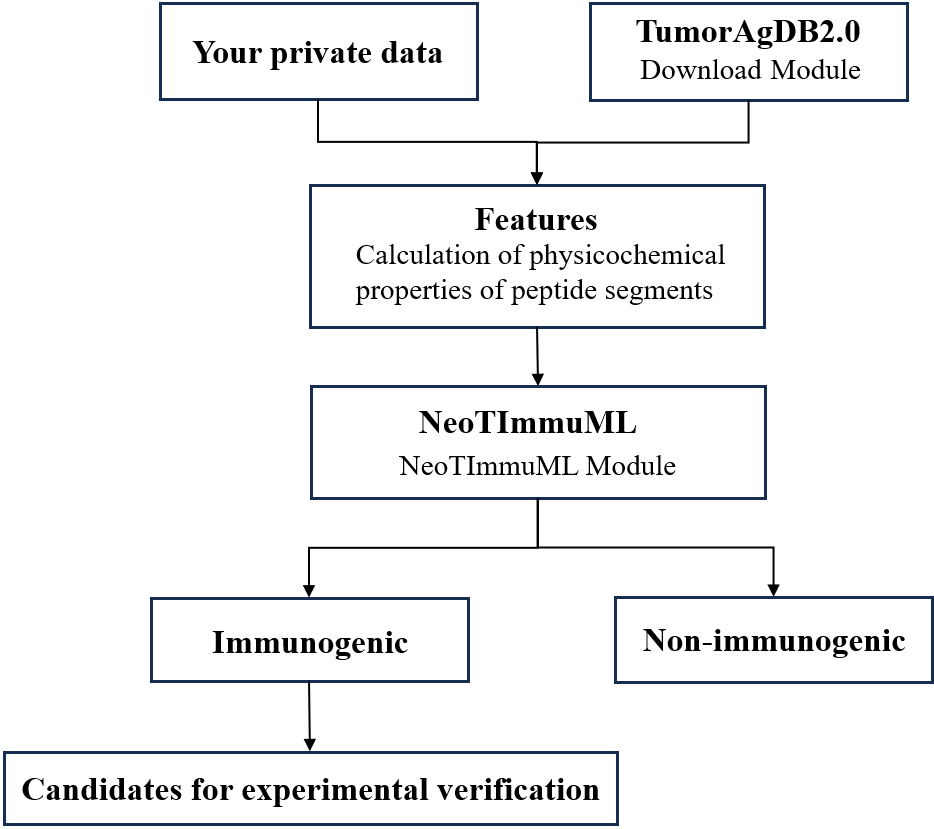

Supplement: Supplementary Figure 1 — Schematic workflow of immunogenicity prediction using TumorAgDB2.0 and NeoTImmuML. Users can utilize their own data or data from the TumorAgDB2.0 database, and select the desired peptide features on the “Features” page to calculate physicochemical properties. Subsequently, our NeoTImmuML tool can be used to predict immunogenicity, and peptides predicted as “Immunogenic” can be considered as candidate peptides for further experimental validation. [file DataSheet1.zip › Supplementary Figure 1.tif]

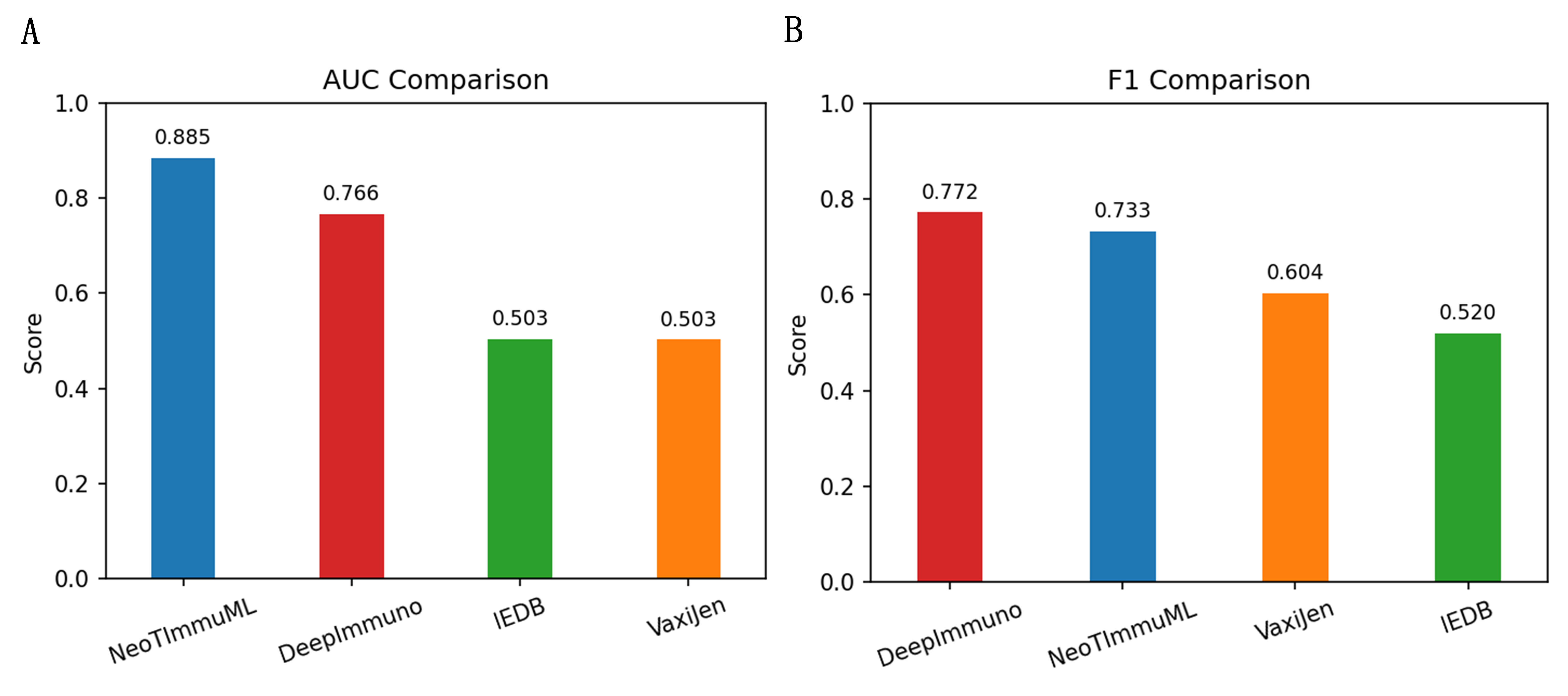

Supplement: Supplementary Figure 1 — Schematic workflow of immunogenicity prediction using TumorAgDB2.0 and NeoTImmuML. Users can utilize their own data or data from the TumorAgDB2.0 database, and select the desired peptide features on the “Features” page to calculate physicochemical properties. Subsequently, our NeoTImmuML tool can be used to predict immunogenicity, and peptides predicted as “Immunogenic” can be considered as candidate peptides for further experimental validation. [file DataSheet1.zip › Supplementary Figure 2.tif]
